# The Association Between Mild Behavioral Impairment‐Apathy Symptoms and Cognitive Symptoms

**DOI:** 10.1002/alz.090525

**Published:** 2025-01-03

**Authors:** Daniella Alicia Vellone, Dylan X. Guan, Zahinoor Ismail

**Affiliations:** ^1^ University of Calgary, Calgary, AB Canada; ^2^ Hotchkiss Brain Institute, University of Calgary, Calgary, AB Canada

## Abstract

**Background:**

Apathy may appear as a less acute late‐life syndrome; however, it is associated with accelerated progression to dementia and contributes to adverse outcomes for patients and caregivers. These findings are not surprising since apathy can cause individuals to forego activities that improve cardiovascular and cognitive health (e.g., exercise), while inadvertently engaging in behaviors associated with greater dementia risk (e.g., social isolation). The objective of this research was to investigate the association between apathy symptoms (in the context of mild behavioral impairment [MBI)]) and cognitive symptoms. We hypothesized that greater MBI‐apathy severity would be significantly associated with greater severity of cognitive symptoms.

**Method:**

All participants enrolled in the Canadian Platform for Research Online to Investigate Health, Quality of Life, Cognition, Behaviour, Function, and Caregiving in Aging (CAN‐PROTECT) aged ≥50 years who had complete MBI‐Checklist (MBI‐C) and Everyday Cognition (ECog‐II) scores were included (n = 1339). Apathy domain scores for interest, initiative, and emotional reactivity were also generated using the six MBI‐C apathy items, and total apathy severity was the sum of all three domain scores. Likewise, ECog‐II domain scores for memory, language, visual‐spatial, and executive function were calculated as the sum of domain items, and total ECog‐II severity was the sum of all domains. Negative binomial regressions (zero‐inflated, if appropriate) assessed associations between total apathy severity and ECog‐II total scores. Domain‐specific analyses were also conducted. Covariates included age, sex, years of education, and non‐apathy MBI (affective dysregulation, impulse dyscontrol, social inappropriateness, psychosis) score.

**Result:**

Across all participants (mean age = 64.5±7.4; 79.5% female), higher MBI‐apathy scores were associated with more severe cognitive symptoms (standardized β [95%CI], 7.2% [4.3‐10.2%]; *p*<0.001). Higher interest (16.5% [9.4‐24.1%]; *p*<0.01) and initiative (18.4% [11.9‐25.4%]; *p*<0.001) apathy domain scores were associated with more severe cognitive symptoms. Total apathy score was also associated with ECog‐II memory (4.9% [2.4‐7.4%]; *p*<0.001), language (5.6% [2.5‐8.6%]; *p*<0.001), visual‐spatial (8.3% [3.3‐13.2%]; *p*<0.01), and executive (10.7% [6.7‐14.7%]; *p*<0.001) domain scores.

**Conclusion:**

These findings provide valuable insight into the complex interplay between apathy and cognitive function, with potential implications for the development of targeted interventions for those affected by apathy‐related cognitive deficits.

